# Structural model of factors contributing to the motivational problem of taking shortcuts at construction workplaces in the Kingdom of Saudi Arabia

**DOI:** 10.1016/j.heliyon.2019.e01220

**Published:** 2019-02-15

**Authors:** Adel M. Al-Shayea, Mohamed Z. Ramadan, Khalid H. Al-Yami

**Affiliations:** a Department of Industrial Engineering, College of Engineering, King Saud University, Riyadh, Saudi Arabia; b Department of Al-Babtain QHSE, Kingdom of Saudi Arabia, Saudi Arabia

**Keywords:** Psychology, Safety engineering, Sociology

## Abstract

An unsafe act is a type of work that involves some risk. Since more than a decade, unsafe act has accounted as a foremost cause of work-related accident, especially in the field of construction. Several attempts have been made to reduce the causes of unsafe acts; however, shortcut, which is a notable type of unsafe act, has received little attention in previous works, even though some of causal effects have been attributed to this habit. This paper aims to reveal an unexplained proportion that can be explained by different underlying causes of motivational problems to shortcuts rather than by habit. Accordingly, two structural models have been proposed based on quantitative and qualitative data from previous reviews associated with the effects on negative feeling and thinking on risk management. These models were tested using on Exploratory Factor analysis (EFA) and Confirmatory Factor Analysis (CFA) on data collected from a cross-section survey conducted at construction sites in largest cities within the Kingdom of Saudi Arabia (KSA), where large number of accidents in these cities occurred. Data of 204 respondents of the mailed questionnaires were analyzed after screening all responses. Statistical Package for Social Sciences (SPSS) (version 22) was used to test for Structural Equation Model (SEM) assumptions. The developed hypotheses were tested and model with the best fit was identified. Findings revealed that 45%–55% of the motivational problems to shortcuts were explained by the selected structural model.

## 1 Introduction

The literature provides a useful account of how the rate of accidents resulting from unsafe acts has remained unchanged for more than a decade. In that period, several researchers have sought to determine the severity of unsafe acts by focusing on human errors. They have reported that human errors cause about 70%–90% of accident-related work injuries (Steve et al., 1995; Berek et al., 2017). One recent publication emphasized the need to focus on the human errors associated with the most common type of accidents that occur at construction sites (Nadhim et al., 2016). They found several reasons for unsafe acts led for construction accidents. Individual characteristics, knowledge, skill, attitude, physical work environment, site conditions, the usability of tools and equipment, training and education, risk exposure, and enhancement of safe work instructions at sites were highlighted as the main causes of human errors. However, the authors stated that the associations between human errors and foremost cause of unsafe acts have not received enough attention in previous works.

A shortcut is an intended act, which is either a routine violation or an expected violation by the performer (Steve et al., 1995; Seo, 2005; Watson et al., 2005; Zin and Ismail, 2012; Yi et al., 2014; Morrow et al., 2014; Oswald et al., 2015). The former constitutes a larger proportion of total unsafe acts, contributing to about 63% of total industrial accidents (Reyes et al., 2015) and about 77% in construction accidents (Darvishi et al., 2015) whereas the latter is rarer (Steve et al., 1995). The habit, worker culture and unavailability of safety equipment were highlighted as the main causes of unsafe behaviors at construction workplace (Darvishi et al., 2015). Continuous training and monitoring were suggested to increase the risk perception toward unsafe behaviors. Risk communication (Moura et al., 2017a,b) and work environment (Jones et al., 2018) were also associated with unsafe behaviors. However, shortcuts-based motivational problems have been highlighted as forefront type of routine violations till today (Steve et al., 1995; Moura et al., 2017a,b; Jones et al., 2018) and the related associations (safety training, follow-up, risk communication and work environment) were not hypnotically and statistically addressed in the those studies.

Hereby, the objective of this paper was to seek to consolidate different measurable indicators of underlying causes to take shortcuts by analyzing quantitative and qualitative data from articles published in the past 10 years as main foundation for constructing the theoretical model. The focus was exclusively on the possible factors associated with the tendency to take shortcuts that could be transmitted through routine perception-based errors. The purpose of this paper was extended to discovering whether the sizes of the causal effects of a mediator could be influenced when stress-based tension moderated the effect of mediation. However, the outputs from this paper cover the unexplained proportion of different underlying factors to the motivational problems to take shortcuts. The proposed models have been confirmed based on evidences collected from participants at construction sites in three major cities within Saudi Arabia.

## 2 Background

### 2.1 Influence of safety training on risk management

Research and speculation on causes of unsafe acts have grown rapidly to identify the relationships between these causes and the probability of unsafe acts. Various studies have highlighted causes such as task complexity, safety practice, safety climate, safety learning, risks perception, safety leadership, and other factors. More than a decade ago, the relationships of safety attitudes with accident rates, risk behaviors and safety climates received attention in addition to the classification of unsafe acts (Siu et al., 2004; Watson et al., 2005). Subsequently, until recently, similar concepts were studied (Darvishi et al., 2015; Remawi et al., 2010; Wang and Sun, 2012; Wachter and Yorio, 2014; Ghahramani and Khalkhali, 2015). In many studies, safety training has been recognized as a vital factor with reference to different aspects of risk management whereas the statistical study of safety training dimensions on risk management has not received sufficient attention.

The safety learn is one of safety training dimensions to gain knowledge, skill and attitude as reported in many researches. More recently, leadership safety training has been reported as a moderator in the relationship between leadership knowledge and risk management (Von Thiele Schwarz et al., 2016) and it was considered as way to prevent construction accidents (Skeepers and Charles, 2015). On the other hand, lack of leadership knowledge on risk management was reported as the reason for the wrong decision made by workers (Ganguly, 2011; Borys, 2012; Amorim and Pereira, 2015). In the field of construction, safety training is highly recommended across different levels of employees toward unsafe acts minimization and safety practice improvement (Garrett and Teizer, 2009; Charehzehi and Ahankoob, 2012; Darvishi et al., 2015).

The reaction and behaviour are two safety training dimensions that have been clearly demonstrated in different researches. The debate about risk perception has gained fresh prominence with many arguing that both understanding and acknowledgment of safe behaviors are subjected to the level of the perception (Agwu, 2012; Amundrud and Aven, 2015). The strength of risk perception can minimize unsafe acts, and it can be achieved by delivering effective safety training to leaders and their followers (Santiago, 2007; Borys, 2012; Leung et al., 2016). When the safety training reflects the life of workers and the reality of their work with reference to the risks they are exposed, it will increase their knowledge about those risks (Floyd and Landis Floyd, 2014; Vasconcelos and Junior, 2015; Biassoni et al., 2015; Ricketts, 2015; Moura et al., 2017a,b). Moreover, it enhances their hazard recognition or risk perception, which in turn, can improve their decision making (Amorim and Pereira, 2015; Skeepers and Charles, 2015; Leung et al., 2016). When the workers receive sufficient amount of safety training, they can complete the assigned work safely (Embrey, 2005) and with minimum errors (Seong et al., 2013). In contrast, poor training was highlighted as one of the significant causes of accidents at construction sites (Garrett and Teizer, 2009; Charehzehi and Ahankoob, 2012), especially when new employees are not trained regarding safe practices (Ganguly, 2011). Hereby, the above findings indicate that both reaction (feeling about risk from the given training program) and behaviour (thinking-based risk management) influence the effectiveness of safety training on risk management. Safety behavior was reported as the function of perceptions regarding risk management (Zhou and Jiang, 2015).

Other researchers highlighted different training dimensions, such as, topic coverage, organizational coverage, and training frequency to identify training as a way to acquire knowledge, skills, and appropriate attitudes (Yi et al., 2014). Training frequency was also reported as another dimension that increases the effectiveness of a given safety training program (Darvishi et al., 2015). The evidence for the need of assessing the effectiveness of safety training to maintain low risk was highlighted by Yi et al. (2014). The safety training is essential for a wide range of safe operations whereas resource constraints and lack of knowledge about training needs are still the major reasons for inadequate training. However, the effectiveness of safety training was evaluated by four aspects, namely, reaction, learning, behavior, and outcomes (Moldovan, 2016). From the above findings, it is evident that there are associations between the effectiveness of safety training and risk management, according to the different levels of employees, which affect their feelings and thoughts about taking risks or managing those risks in a safe manner. In this paper, five indicators have been used based on above findings to infer about the effectiveness of safety training on risk management. Frequency, coverage, reaction, learn and behavior were used to indicate about safety training effectiveness in order to examine the statistical influence on both feeling and thinking toward risk management. Unlike other studies, this paper intends to examine the effect of safety training statistically through a maximum possible indicators.

### 2.2 Influence of stress-based tension on risk management

Despite the importance of tension in the work environment, there remains a lack of statistical evidence on its association with the motivational problem of taking shortcuts. It was emphasized that rules and procedures would not lead to risk management because of the weak safety perception while communicating their contents (Sheridan, 2008; Shi and Inoue, 2012; Moura et al., 2017a,b; Liang et al., 2018). In a recent publication, the lack of ability to transfer knowledge into effective actions was highlighted (Moreno and Cavazotte, 2015; Jepson et al., 2017; Moura et al., 2017a,b; Liang et al., 2018; Oah et al., 2018). The authors also recommended that knowledge management is a necessary factor to support employees' decision making while performing their tasks. The visual-based design of safety signboards or posters as communication media to recognize the surrounding hazard and perceive the degree of risk consequence (Amorim and Pereira, 2015). Moreover, the same advice was highlighted in a more recent study on information design (Yoon et al., 2016). The above authors emphasize the importance of communication as a source of maintaining proper risk management and acknowledge that it could be a source of tension if it is not rational (Jepson et al., 2017; Oah et al., 2018).

Researchers have paid attention to the physical work environment by focusing on the commitment to keeping safety as the first priority. The normal working condition was significantly associated with the level of safety compliance. The latter was affected when the former interacted with issues related to inappropriate physical environments (Leung et al., 2010; Moura et al., 2017a,b; Techera et al., 2018). It was a result of unavailability of risk management procedure to control those effects in the earlier stage (Bowen et al., 2014; Leung et al., 2016). The ability to complete a task, positive expectation, self-learning to achieve a task, and ability to manage work-related risks were identified as factors that were positively associated with the perception of the physical work environment (Bergheim et al., 2015; Moura et al., 2017a,b). The work environment increases the possibility of misunderstandings related to safety perception (Ganguly, 2011; Blazsin and Guldenmund, 2015; Liang et al., 2018). The above evidences emphasizes the negative aspects of the physical work environment that can act as another source of tension.

Few studies aimed to understand the constraints on workers' capabilities and their needs based on the conditions of their work environment (Report et al., 2001; Jaffar and Lop, 2011a,b; Moura et al., 2017a,b; Techera et al., 2018). Another study indicated that the effect of safety compliance on safe work practices was predicted by the effects of both workload and extra rules (Mohammed et al., 2016; Leung et al., 2016). These studies confirmed the need to address the margin of a personal lifetime to serve the important life-issues and minimize the associated tension. Duration of work was reported as a source of risk when it causes tensions (Hobbs and Williamson, 2003; Leung et al., 2016). Additional evidence for maintaining enough margin of a personal lifetime was explained when tight schedule contributed to taking shortcuts (Zhou and Jiang, 2015). The work procedure was indicated as a source of risk when it was not developed appropriately based on the available resources in the physical work environment (Praino and Joseph, 2016). The risk management was also addressed for the available time when it was less than that required for completing the work. However, risk communication, physical work environment and the margin of a personal lifetime were used in this paper as new subject to infer about the statistical influence of the stress-based tension as a predictor for both feeling and thinking toward risk management.

### 2.3 Influence of routine perception-based errors on risk management

Several studies have explored different factors influencing the safety perception. More recently, the lack of hazard recognition or risk perception was reported as the reason for the wrong decisions of workers (Amorim and Pereira, 2015; Ozmec et al., 2015; Moura et al., 2017a,b). Risk assessment is used to increase risk perception (Agwu, 2012) if the risk is understood, recognized (Rasmussen and Lundell, 2012; Amundrud and Aven, 2015), and ultimately controlled (Sheridan, 2008; Leung et al., 2016). Specifically, safety commitment/communication, safety involvement/training, positive safety practice, safety competency, safety procedure, responsibility, supportive environment, and safety prioritization were statistically associated with the routine safety perception (Ghahramani and Khalkhali, 2015; Moura et al., 2017a,b). Thus, feelings about stimuli experienced at the workplace will affect the thoughts pertaining to risk management.

Safety behavior significantly affected the safety perception in the construction field when both work environment and workers' needs existed (Pecquet, 2013; Liu and Song, 2014). The interactions between work behaviour and capability were affected the risk management and consequently led to unsafe decision (Mitropoulos et al., 2010). When the task does not fit with individual, the likelihood of routine safety violation will increase (Jaffar and Lop, 2011a,b) and mostly taking shortcuts (Jones et al., 2018). Emotional risk perception can be achieved when risk communication, proper safety program, good work environment, minimum workload are controlled (Oah et al., 2018). The above publications indicate different issues associated with feeling-based risks and thinking-based risks. Unlike other publications, those issues were statistically examined to infer about routine perception-based errors.

Risk communication can improve safety perception and minimize unsafe acts (Zhou and Jiang, 2015). Further, the perception of role clarity, career commitment, and job performance was statistically predicted by risk communication (Borys, 2012; Kalkavan and Katrinli, 2014; Moura et al., 2017a,b). The perception of near misses is one of the essential practices in risk communication to minimize the unsafe acts/conditions. An attention statistically mediated the relationship between safety behavior and the combination of attitude, personal norms, and perceived control (Watson et al., 2005; Santiago, 2007). The above findings support the importance of the role of feelings and thoughts regarding risk management in the incidence of routine perception-based errors.

### 2.4 Motivational problem of taking shortcuts

Researchers have paid significantly less attention to the factors contributing to the motivational problem of taking shortcuts. Safety rule violations were associated with time pressure (Hobbs and Williamson, 2003). The issue of time pressure has been a controversial and much-disputed subject within the field of safety violation. Hence, the relationship between task complexity, experience, time availability, and time pressure were studied as predictors of the probability of human error (Nummenmaa et al., 2014). The systematic understanding of how the time pressure contributes to the motivational problem of taking shortcuts can be inferred by the margin of a personal lifetime, which is considered as one of measurable indicators of stress-based tension in this paper.

The risk of taking shortcut can be increased when workers seek for positive value by ignoring safety regulations (Zhou and Jiang, 2015). The effect of emotional risk communication can make positive changing on the worker's behavior (Kalkavan and Katrinli, 2014; Bowen et al., 2014; Hayes, 2015; Jepson et al., 2017; Oah et al., 2018). Different fatigue-related issues were highlighted in construction sites as incomplete recovery or lack of sufficient time after work, workload, work environment and social environment (Techera et al., 2018). They found those issues have a strong association with the construction accidents. Risk knowledge was highlighted as a way to have proper decision (Aven, 2016) and it can be achieved through safety training (Jepson et al., 2017). The above findings support three indicators (saving time, reducing effort, and gaining value) that contribute to the motivational problem of taking shortcuts as newly represented by this paper.

### 2.5 Anticipated theory on the motivational problem of taking shortcuts

Safety training effectiveness is an exogenous factor that can be inferred by different indicators (frequency, coverage, reaction, learning, and behavior) that combined from the previous reviews (Santiago, 2007; Garrett and Teizer, 2009; Ganguly, 2011; Agwu, 2012; Charehzehi and Ahankoob, 2012; Borys, 2012; Moldovan, 2016; Floyd and Landis Floyd, 2014; Liu and Song, 2014; Amorim and Pereira, 2015; Vasconcelos and Junior, 2015; Darvishi et al., 2015; Zhou and Jiang, 2015; Biassoni et al., 2015; Ricketts, 2015; Skeepers and Charles, 2015; Amundrud and Aven, 2015; Leung et al., 2016; Von Thiele Schwarz et al., 2016; Moura et al., 2017a,b).

Stress-based tension is another exogenous factor. It is affected by communication, physical work environment, and margin of a personal lifetime based on the evidences summarized in the literature section (Report et al., 2001; Hobbs and Williamson, 2003; Sheridan, 2008; Leung et al., 2010; Jaffar and Lop, 2011a,b; Ganguly, 2011; Shi and Inoue, 2012; Bowen et al., 2014; Amorim and Pereira, 2015; Blazsin and Guldenmund, 2015; Zhou and Jiang, 2015; Bergheim et al., 2015; Moreno and Cavazotte, 2015; Praino and Joseph, 2016; Leung et al., 2016; Mohammed et al., 2016; Yoon et al., 2016; Jepson et al., 2017; Moura et al., 2017a,b; Liang et al., 2018; Oah et al., 2018; Techera et al., 2018).

Routine perception-based error is an endogenous factor that is inferred from the feeling-based and thinking-based risks about stimuli experienced at the workplace and the subsequent actions taken (Watson et al., 2005; Santiago, 2007; Sheridan, 2008; Mitropoulos et al., 2010; Jaffar and Lop, 2011a,b; Borys, 2012; Pecquet, 2013; Liu and Song, 2014; Kalkavan and Katrinli, 2014; Ghahramani and Khalkhali, 2015; Amorim and Pereira, 2015; Zhou and Jiang, 2015; Ozmec et al., 2015; Leung et al., 2016; Moura et al., 2017a,b; Oah et al., 2018). The cycle of routine perception-based error begins from feeling to understand the consequence of the observed risk. Then, the cycle continuous to evaluate the risk and take the action. The action will be perceived by another person and the same cycle is repeated. This fact was concluded based on evidences in the literature (Agwu, 2012; Rasmussen and Lundell, 2012; Amundrud and Aven, 2015).

**Fig. 1:**
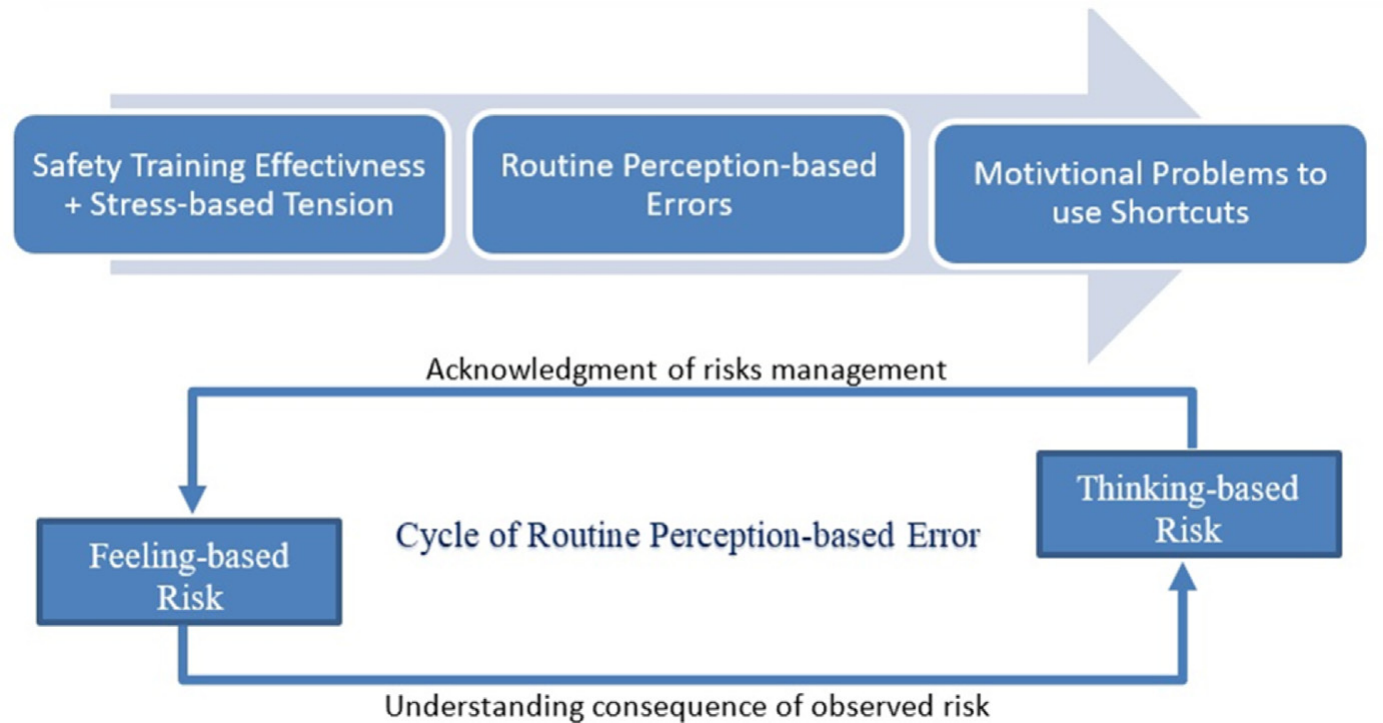
Overview of suggested theory on motivational problem to shortcut and it is developed based on supplied information from literature.

**Fig. 2:**
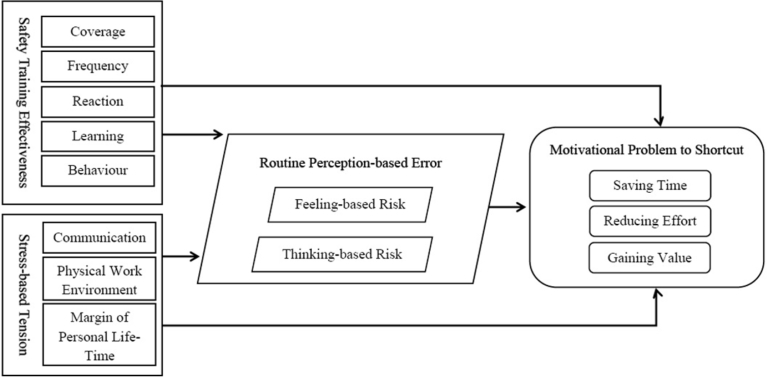
Details of basic conceptual model as theoretical framework developed from literature findings.

The motivational problem of taking shortcuts is the main endogenous factor in the present study. It was inferred by three issues, namely, saving time, reducing effort, and gaining value that were identified from the collected evidences (Hobbs and Williamson, 2003; Nummenmaa et al., 2014; Kalkavan and Katrinli, 2014; Bowen et al., 2014; Zhou and Jiang, 2015; Hayes, 2015; Aven, 2016; Jepson et al., 2017; Oah et al., 2018; Techera et al., 2018). Hereby, the literature was used as a foundation for constructing the planned theory can be expressed as a function of safety training effectiveness, stress-based tension, and routine perception-based errors that motivate the use of shortcuts, as shown in Fig. 1 and in details as shown in Fig. 2.

### 2.6 Specifications of the theorized basic structural model

The aforementioned exogenous factors (safety training effectiveness and stress-based tension) were assessed as independent effects in the basic structural model presented in this study. In the proposed theory, these exogenous factors are associated with routine perception-based errors, which in turn, cause the motivational problem of taking shortcuts. Each factor is subjected to different measurable indicators as presented in Fig. 2.

### 2.7 Specifications of the theorized alternative structural model

**Fig. 3:**
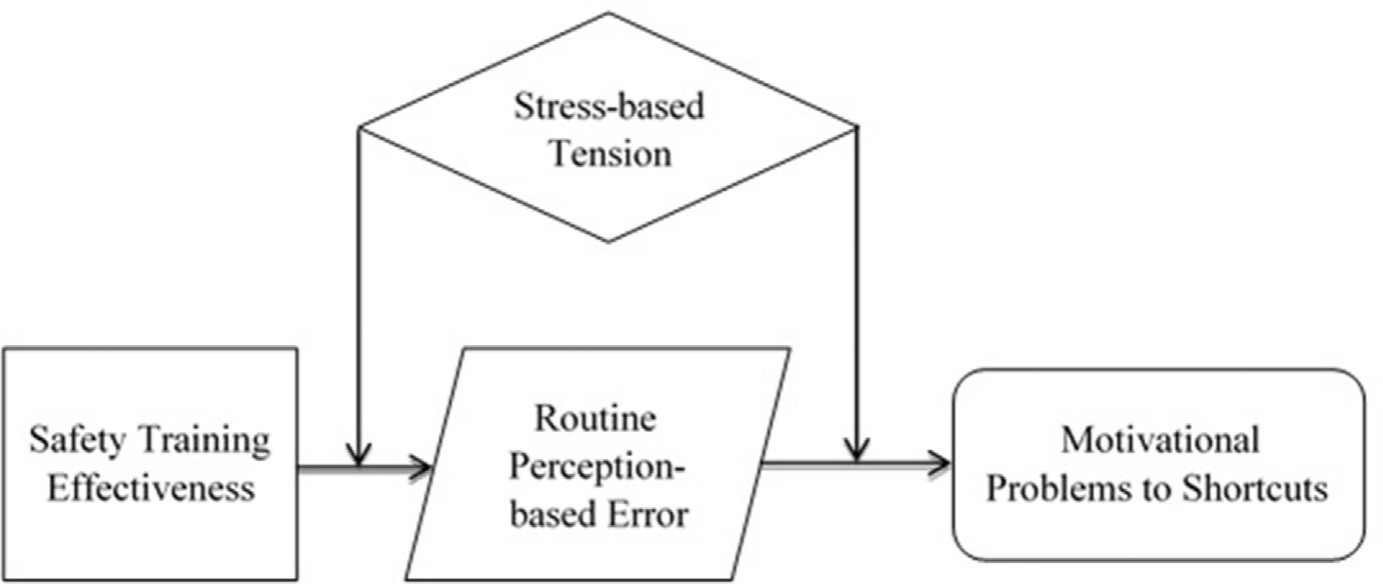
The alternative conceptual model having moderated mediation effects.

Based on the above literature, communication and poor work environment are considered to increase the possibility of misunderstanding safety perceptions (Ganguly, 2011; Blazsin and Guldenmund, 2015; Liang et al., 2018). These findings indicate the effect of stress-based tension that can moderate the relationship between the effectiveness of safety training and the motivational problem of taking shortcuts via routine perception-based errors. The theorized moderating effect was employed to examine the strong influences of the relationship in the mediating effect, called as the moderated mediation effect (Pezeshki et al., 2017) and it can be represented as shown in Fig. 3.

### 2.8 Hypotheses of the theorized structural models

The previous reviews were support the development of two models as shown in Figs. 2 and 3, hereinbefore. In those figures, there are eight different hypotheses were expressed. Safety training effectiveness (STE) enhances the feelings and thoughts regarding right safety practices and minimizes the chances of being at risk. The routine perception-based error (RPBE) accommodates both positive feelings and thoughts to understand the received stimuli and acknowledge the decided action. Therefore, the following hypotheses were proposed:

H1 STE has a direct positive impact on RPBE.

H2 STE has a direct negative impact on the motivational problem to shortcut (MPS).

H3 RPBE has a direct negative impact on MPS.

H4 The effect of the causal path between STE and MPS is transmitted through RPBE.

Stress-based tension (SBT) is a negative or a positive stimulus received from the workplace from the nature of the communication and physical environment at the workplace, and the existence of necessary life-issues. Therefore, the following hypotheses were proposed:

H5 SBT has a direct negative impact on RPBE.

H6 SBT has a direct positive impact on MPS.

H7 The effect of the causal path between SBT and MPS is transmitted through RPBE.

The alternative model demonstrates that stress-based tension can act as a moderator instead of an independent factor. Hence, it was hypothesized that:

H8 The causal path between STE and MPS via RPBE could be influenced by a change SBT.

## 3 Materials and methods

Different search engines were employed in search process, such as, Safety Science, Reliability of Engineering and System Safety, Accident Analysis and Prevention, Safety and Health at Work, Human Factors and Ergonomics Society, International Journal of Environmental Research and Public Health, Journal of Construction Engineering and Management, Journal of Management in engineering and Procedia Engineering. Moreover, there are different search terms were used for selection process, such as, unsafe acts, intention unsafe acts management, effective safety training to shortcuts, risk perception, risk management, worker tension and accidents and violation-based accident. Based on the scope of this paper, 368 publications were identified (322 articles, 8 theses, 4 conferences, 7 books, and 28 post-notes). The post-notes were published on a professional safety website. Two filter techniques were used to collect all the information relevant to the objective of this paper. The first filter was carried out based on the contents of abstracts, while the second filter was conducted after reviewing the contents of the whole document. The first filter yielded 204 publications (182 articles, 3 theses, 2 conferences, 4 books, and 13 post-notes), while the second led to 170 articles, 1 thesis, 4 books, 2 conferences, and 6 post-notes). During summarizing the collected pieces of evidence from the main body of each research, the final number of articles was fixed at 58 articles in addition to other types of publications that met the research criteria. The findings from this stage were analyzed to configure the main foundation for constructing the proposed structural models.

Previous studies have provided evidence for the theory proposed in this paper and they supplied sufficient information to design the research instrument since the required data were not available. Hence, a mailed questionnaire was designed according to specifications, in study made by Nadhim et al. (2016), to assess all measurable indicators in the theorized model. The developed mailed questionnaire contains items that are rated on a 5-point Likert scale. For further details, see the questionnaire in supplementary material file. The appropriateness of the tool was confirmed through a pretest survey and pilot test conducted in the most high-risk sectors in KSA.

First, the pretest survey was conducted, with a response rate of 73.33% (42.86% academics and 100% consultants). After modifying the mailed questionnaire based on the feedback received through the pretest survey, the validity assessment was proceeded according to procedures documented by Ghahramani and Khalkhali (2015) as well as Pezeshki et al. (2017). The validity was assessed in two ways; using the response on content validity (CVR) and the content validity index (CVI). The criteria used for assessing the validity it this paper were CVI ≥ 0.75 and CVR similar to table reported by Colin and Andrew in 2014. Four aspects were assessed in the pretest survey (relevance, clarity, simplicity, and smoothness of contents and necessity) to adapt and adopt the validity assessment based on the pretest feedback. The criteria used for assessing the reliability (α) was α > 0.7, as adopted by most researchers. Both validity and reliability assessments were confirmed using these criteria.

The collected data was limited to construction sites (which accounted for 46% of the total accidents in KSA) in the largest cities (Riyadh, Dammam, and Jeddah) in which 43% of the total accidents had occurred according to the updated accidents records in 2016 (information supplied from open data in General Organization of Social Insurance as listed in reference). In total, 47 construction companies within these cities responded to the mailed questionnaire. The pilot test survey was then conducted with candidates from the main sample in this paper (a group of employees from construction sites within Riyadh, Dammam, and Jeddah). The planned sample was 70, which was higher than the recommended sample range of 30–50 according to guidelines presented by Nadhim et al. (2016), for a pilot test. However, the response rate was 87.14% (52.46% respondents worked at different construction sites in Riyadh, 36.07% respondents were from Dammam, and 11.47% respondents were from Jeddah). Most participants were aged 30–42 years, and most of them had an educational level of less than a diploma (high school, intermediate school, and primary school). Few participants (17%) had more than 10 years' experience in the construction field and most of them had less than 5 years. A reliability assessment was conducted to verify the consistency of responses between respondents and it was confirmed based on the criterion mentioned earlier (α > 0.7).

After assessing the validity and reliability of research instrument through the pretest and pilot test procedures, the mailed questionnaire was sent to 280 workers at the 47 construction sites. This sample is greater than the recommended sample size that was calculated according to a free statistics online calculator (link of free online calculator is available in references list). Though the response rate was 97.2%, the effective response rate was 82.4% after applying a horizontal analysis for every individual groups to identify missing responses and the unengaged respondents. Previous studies have often reported a response rate of 44.5% (Morrow et al., 2014) and 48% (Schreiber et al., 2006) when using the same SEM technique in the same environmental field. However, the characteristics of the respondents in the main survey validate the randomness of sampling of participants from different clusters (Riyadh, Dammam, and Jeddah). Most of the participants were aged from 30 to 42 year. Their experience levels varied greatly, and surprisingly, older participants tended to have an experience of less than ten years. Further, 41% of the respondents had an experience of over 5 years and 45% had an experience of 1–5 years. Few of the workers were new (14% of the total participants), and their responses were also taken into consideration. Most participants fell under the job category of “workers” (89%), and this job category was one of the major focus areas in this paper as they worked at the site for the longest. The remaining participants were site supervisors (5%) and site safety inspectors (6%) who worked in the same environment and dealt with accidents records. Moreover, few participants had qualifications higher than a diploma certificate (11%), while majority of them (64%) had an educational level lower than a diploma. This indicates the need for safety training for construction workers, as highlighted in the existing literature. For further information to questionnaire and data, please see Supplemental File.

The collected data were tabulated in SPSS (version 22) for checking the assumptions of structural modeling (SEM), and explained in the next section. An Exploratory Factor analysis (EFA) and Confirmatory Factor Analysis (CFA) was conducted by applying the criteria of model fit indices and modification strategy as highlighted by Schumacker et al. (2010) as well as Howard, 2013. Then, the Maximum Likelihood (ML) estimation was carried out in AMOS (Analysis of Moment Structures; version 25) to examine both structural models and to confirm the hypotheses. The mediating effects between the casual paths of latent factors were examined using the common procedure mentioned by Che et al. (2017), in addition to the Sobel test statistic (Z-score) for testing the significant mediation, conducted using a free statistics online calculator. The significant moderator was also confirmed through the available worksheet for plotting interaction terms as well as a free statistics online calculator for examining the significant moderator.

This research was approved by the ethics committee of King Saud University in March 2016. Each participant received a questionnaire with cover page that contains the rights, the purpose of the study, the procedures, the potential risks and benefits of participation. At the end of questionnaire, he asked to check a designated box on the questionnaire for the value of the study to him.

## 4 Results

### 4.1 Hypotheses verification

The conceptual models were subjected considerable hypotheses as aforementioned. The direct casual paths and mediations were verified to confirm the developed hypothesis based on the procedure used by Che et al. (2017) and moderated mediation verification as represented by Awang (2012). Unstandardized regression outputs were used to report estimated values and significant level for each causal path as shown in Table 1, heretofore. The results of hypotheses verification explain in the same table.

**Table 1:** Hypotheses verifications.

Path	Estimate	P-value	Result of Hypothesis (H)
STE → RPBE	0.64	Sig.	Positive association and H1 significantly confirmed the direct causal path.
STE → MPS	-0.356	Sig.	Negative association and H2 significantly confirmed the direct causal path.
RPBE → MPS	-0.087	Sig.	Negative association and H3 significantly confirmed the direct causal path.
STE → MPS STE → RPBE RPBE → MPS	-0.531 0.641 0.279	Sig. Sig. Sig.	All causal paths were still significant after adding RPBE as mediator and direct causal path was slightly improved. It confirmed partial mediation and H4 was considered valid.
SBT → RPBE	-0.727	Sig.	Negative association and H5 significantly confirmed the direct causal path.
SBT → MPS	0.355	Sig.	Positive association and H6 significantly confirmed the direct causal path.
SBT → MPS SBT → RPBE RPBE → MPS	0.365 -0.728 0.28	Sig. Sig. Not Sig.	Causal paths were significant after adding RPBE as mediator except on path. Hence, H7 was not supported.
STE → RPBE RPBE → MPS	0.388 0.233	Sig. Sig.	The moderated mediation confirmed since the size of indirect effects were changed with different level of SBT.

### 4.2 Model confirmation

Screening data were employed in SPSS to verify the assumptions for using structural equation modeling (SEM). Model specifications were assessed using an Exploratory Factor Analysis (EFA) after confirming the SEM assumptions. The EFA was used to confirm the number of factors in different observations (measurable indicators). The outputs of the rotated factor matrix have been summarized in Table 2, which also shows the number of factors and their loading indicators.

**Table 2:** Rotated factor matrix of the Exploratory Factor Analysis.

Indicators	Factors and Related Loading Indicators			
	STE	SBT	RPBE	MPS
STEF	.625			
STEC	.837			
STER	.820			
STEL	.821			
STEB	.786			
SBTC		.919		
SBTE		.930		
SBTP		.636		
RPBEF			.882	
RBPET			.900	
MPSST				.924
MPSRE				.923
MPSGV				.910

**Fig. 4:**
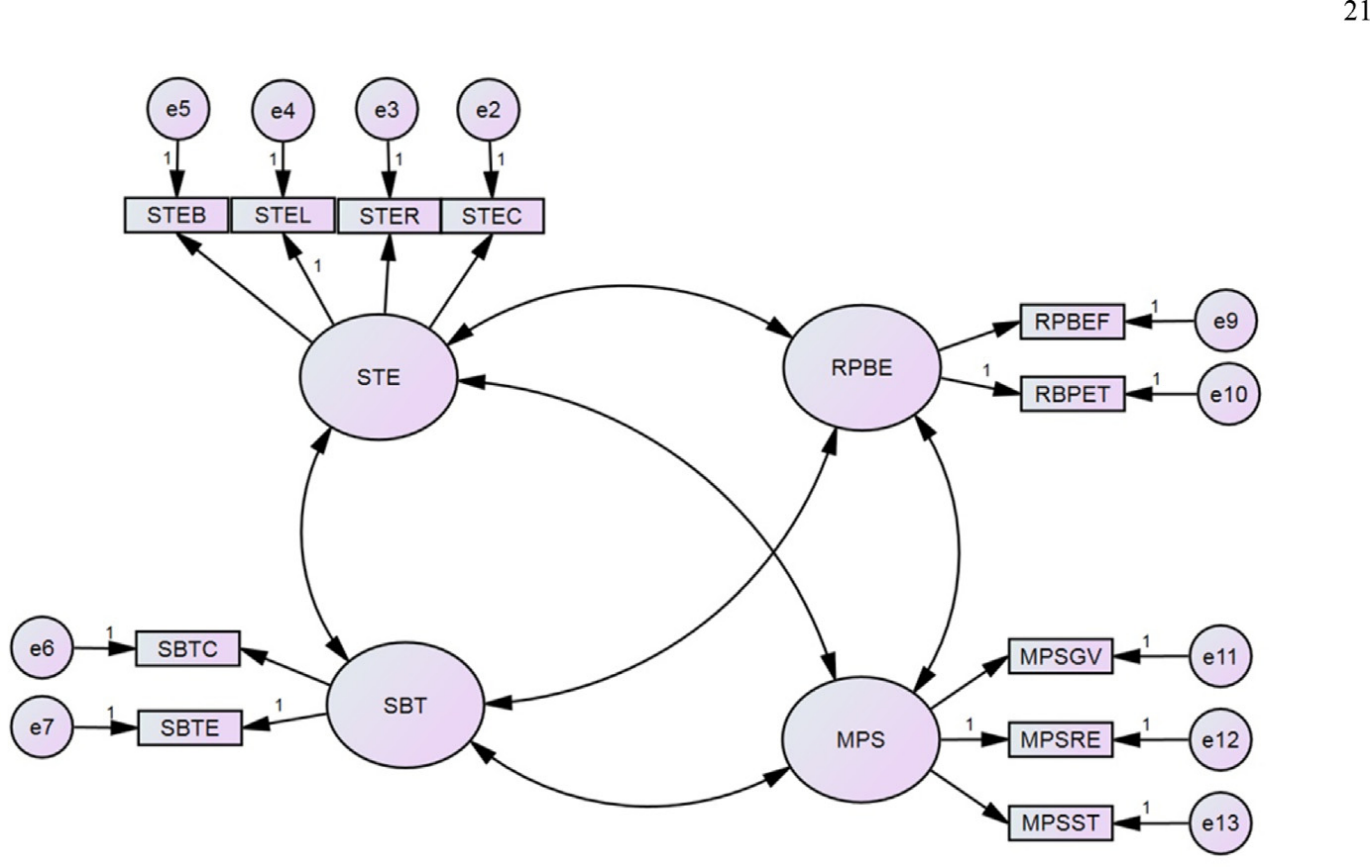
Development of the basic model for the Confirmatory Factor Analysis.

AMOS was used to confirm these validities by connecting each latent factor to every other latent factor by a covariance (or correlation) pathway prior analysis, as shown in Fig. 4. This figure also shows the final measurement model after reaching good fit indices (GFI = 0.94, NFI = 0.96, CFI = 0.99, and RMSEA = 0.7). These indices were reasonably accepted. Frequency and margin of personal lifetime were excluded to achieve a good fit in the measurement models.

### 4.3 Estimation of the basic model and testing the related hypotheses

**Fig. 5:**
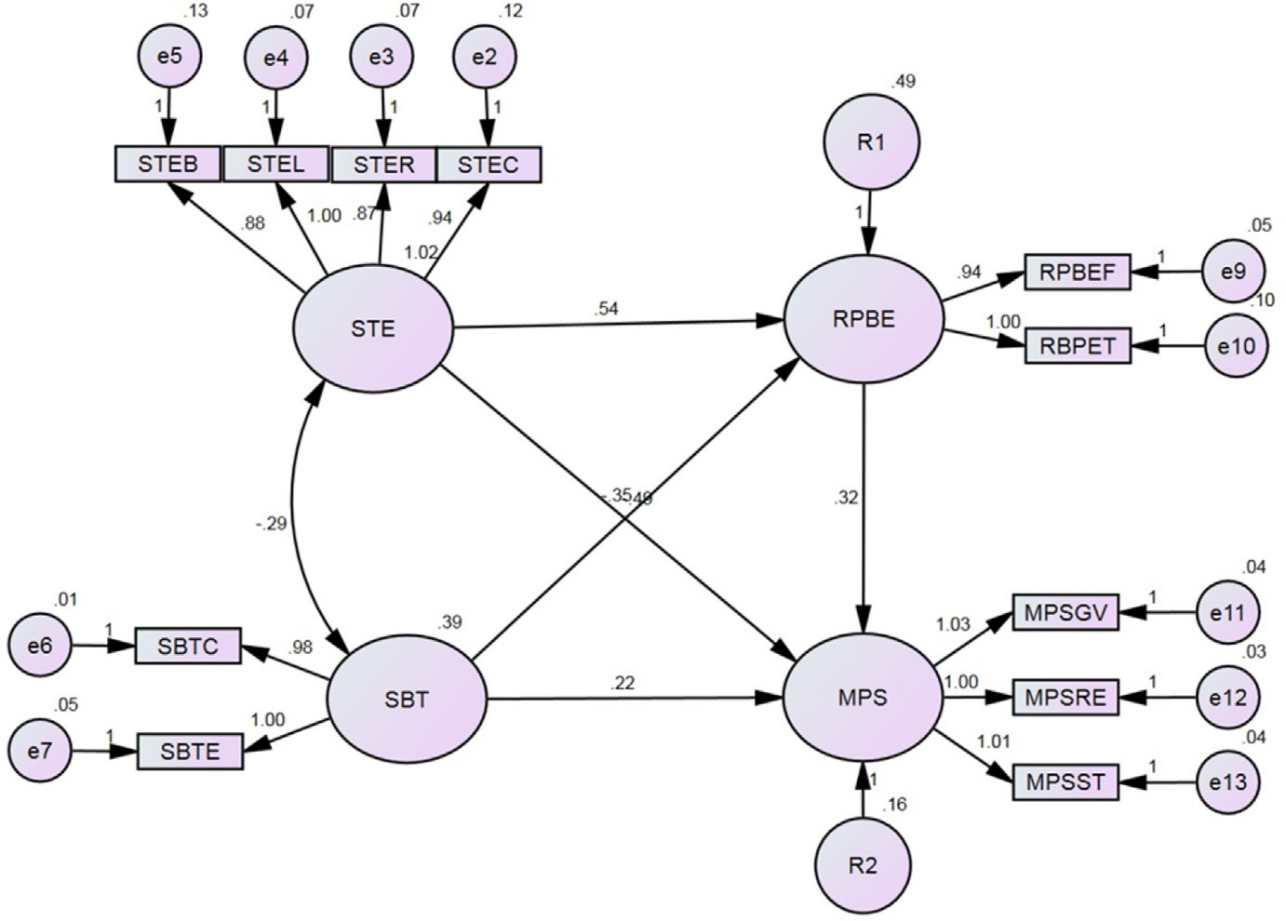
Unstandardized regression weights of the estimated basic structural model.

Maximum Likelihood (ML) estimation was carried out in AMOS to examine the basic structural model after connecting the casual paths of latent factors and adding disturbances. Fig. 5 presents the outputs after running ML for the unstandardized estimates to assess the significant prediction between latent factors. However, GFI (0.94), CFI (0.99), NFI (0.96), and RMSEA (0.07) were found same as previous indications of CFA assessment for goodness of fit. From ML outputs, all causal paths between the latent factors were significantly estimated at a 95% confidence level based on the observed indicators represented in the structural model. The standardized residual covariances were found within the acceptable range of standard deviations (±2). The sub-models were used to test the hypotheses of direct causal paths. Unstandardized regression outputs were used to report the estimated value and significance of each causal path. The standardized regression outputs were used to report the statistical proportion of variance explained by exogenous factor. Table 3 shows the output of the estimated direct causal paths.

**Table 3:** Outputs of the estimated direct causal paths in the model.

Path	Estimate	P-value	R^2^	GFI	NFI	CFI	RMESA
STE → RPBE	0.541	Sig.	32%	0.976	0.991	0.994	0.089
STE → MPS	-0.495	Sig.	73%	0.967	0.987	0.993	0.081
SBT → RPBE	-0.353	Sig.	5%	0.999	1.0	1.0	0.0
SBT → MPS	0.220	Sig.	5%	0.985	0.993	0.997	0.071
RPBE → MPS	0.323	Sig.	29%	0.992	0.996	1.0	0.022

From Table 3, the developed hypotheses of the direct causal paths were tested and summarized as follows:

• It was confirmed that STE significantly and positively affected RPBE and the latter was statistically explained by 32% of the change in one unit of the standard deviation of the former. The results supported the related hypothesis and showed a good model fit of the hypothesized direct causal path.
• It was also confirmed that STE significantly and negatively affected MPS and it statistically explained 73% of the change in the latter. These results also supported the related hypothesis and shown a good model fit.
• It was confirmed that SBT significantly and negatively affected RPBE and explained 5% of the variation in the latter. These results supported the related hypothesis with a good model fit.
• It was also confirmed that SBT significantly and positively affected MPS and explained 5% of the variation in the latter. These results supported the related hypothesis with a good model fit.
• Finally, it was confirmed that RPBE significantly and positively affected MPS and explained 29% of the variance in the latter. These results supported the related hypothesis with opposite direction and the direct causal path was a good fit.

However, the proportion of variance was affected by the size of each factor (number of measurable indicators and both macro and micro, intra-indicator and inter-indicator variations). Based on the common procedure for testing a mediator, the following was confirmed:

• Both STE and SBT had significant direct effects on MPS before adding RPBE as a mediator. After adding RPBE, the direct causal paths (STE→MPS and SBT→MPS) were statistically significant and the values listed in Table 2 reduced to -0.495 and 0.22, respectively. Hence, RPBE was partially mediated the direct causal paths. Surprisingly, RPBE was significantly and partially mediated the causal path between SBT and MPS when the full model was tested unlike previous result in hypotheses verification.
• Both STE and SBT had a significant direct effect on RPBE.
• RPBE had a significant direct effect on MPS.

The above investigations of RPBE as a mediator between STE, SBT, and MPS were theoretically verified by comparing the calculated Z-test score with the critical condition (-1.96 < Z < 1.96). A free statistics online calculator was used and a value of 5.70 was obtained when RPBE mediated the casual path between STE and MPS. It was -3.32 when the effect of the casual path between SBT and MPS was mediated by RPBE, but these Z-scores were out of the aforementioned critical condition. Therefore, the Sobel test statistic was used to confirm that RPBE significantly mediated the causal relationship between STE and MPS, as well as that between SBT and MPS.

### 4.4 Estimation of the alternative model and testing the related hypotheses

**Fig. 6:**
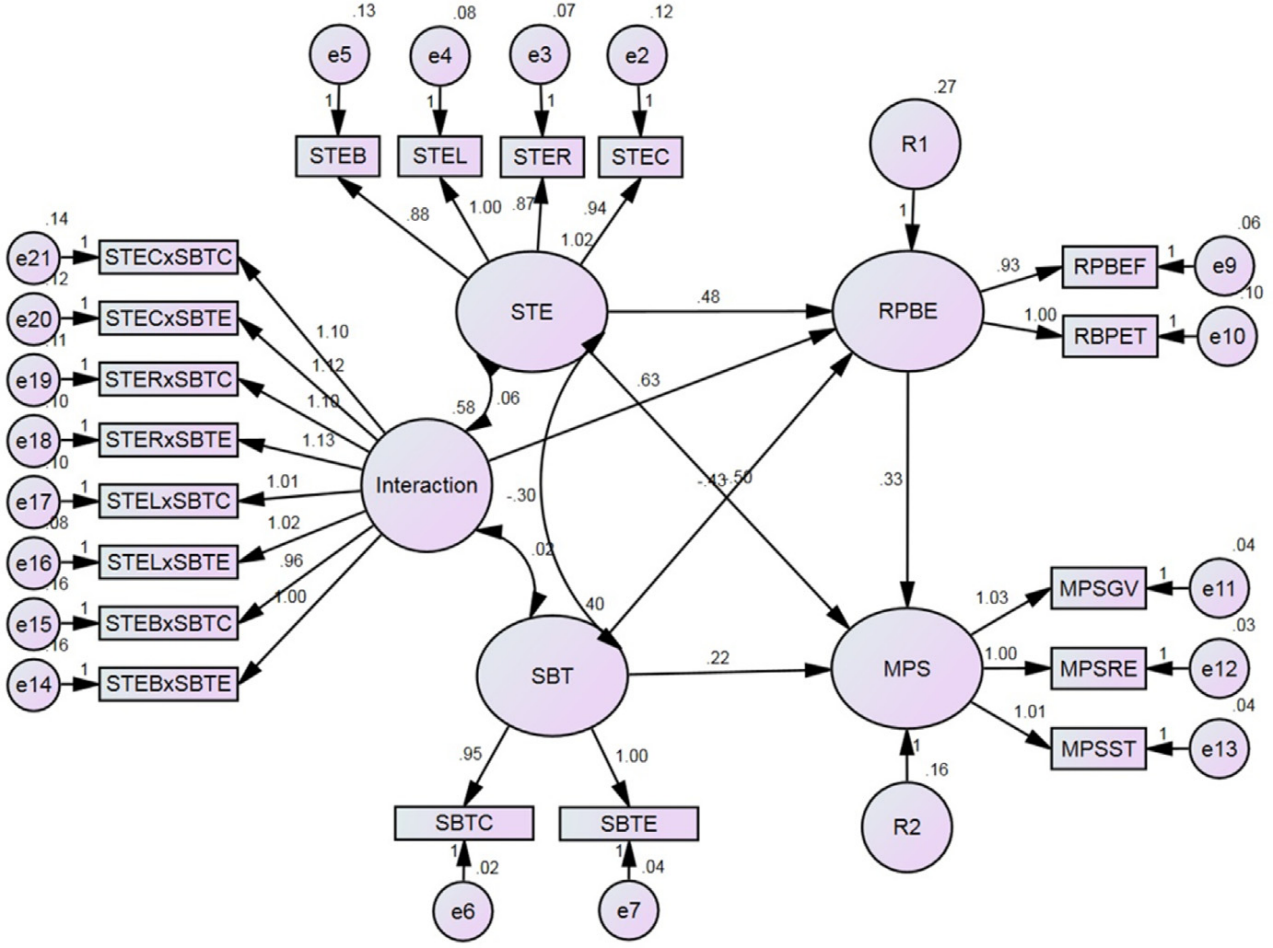
Graphical representation of model with interacted latent factor.

The estimation process of the moderating effects based on a continuous latent factor was assessed based on the significant difference between the slopes of two interacting terms. In this situation, the interactions between SBT and STE that affected the causal paths between STE and MPS via RPBE were examined. Data were primarily prepared in SPSS by computing the interacted indicators and saved as standardized interaction terms. Then, these indicators were uploaded into AMOS as members of the new family (new exogenous factor) and ultimately regressed with both RPBE and MPS. Additional, the related exogenous factor (SBT) was also connected with a regression line to the same endogenous factors (RPBE and MPS) as shown in Fig. 6. ML estimation was also performed to examine the alternative structural model with interaction (SBT × STE). The interaction was statistically significant with RPBE and nonsignificant with MPS (0.058 marginally exceeded the p-value cutoff of 0.05).

**Fig. 7:**
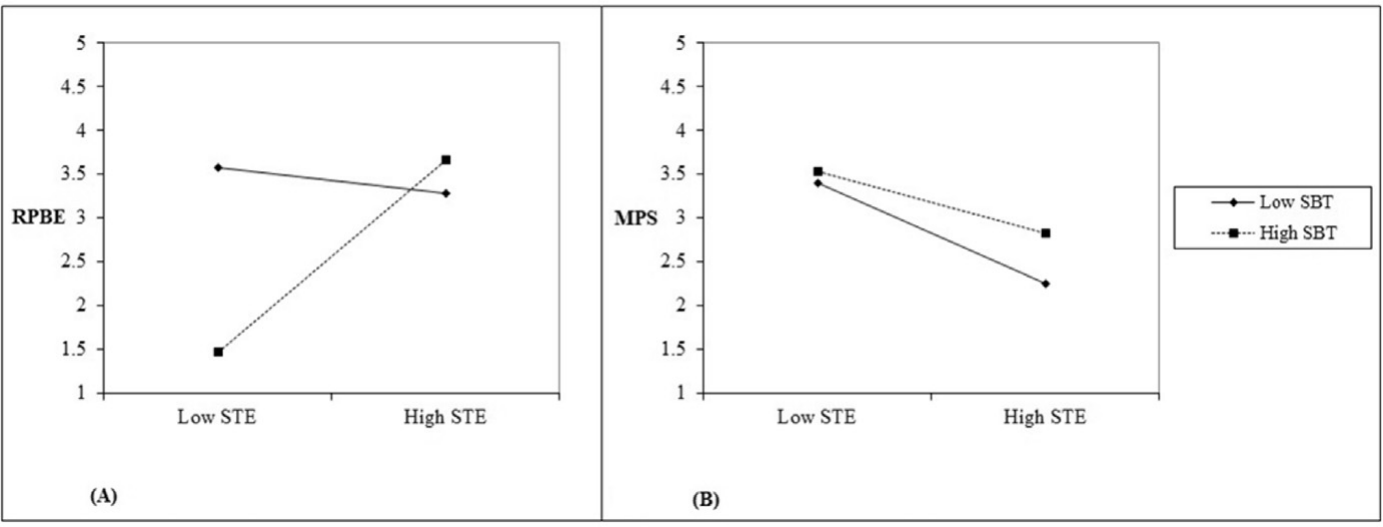
Interaction in causal path between: A) STE and RPBE comparison with B) STE and MPS.

Additional investigation was carried out by using the available online worksheet that was designed to plot the two-way interaction effects for unstandardized regression weights. Fig. 7 shows that both lines show a good sign of interaction in the causal path between STE and RPBE, whereas the collected data was not supported another interaction. Moreover, an online Free Statistics Calculator was used to prove the existence of significant moderator in the causal paths. The calculated t-values were statistically significant for both casual paths. However, GFI (0.77), CFI (0.84), NFI (0.83), and RMSEA (0.195) remained unchanged. Hereby, the alternative structural model did not exhibit adequate fit.

The last objective of this paper is to discover whether the casual effects of STE on MPS via RPBE differ based on the effect of SBT. Based on specifications of Hayes, the moderated mediation occurs when one of the following three approaches exists: the nature of mediation, size of indirect effects, or direction of indirect paths should exist during assessing both mediators before and after adding a moderator. The comparisons between both situations with reference to these three angles have been presented in Table 4. The overall results observed unchanged with reference to the nature of mediation and the direction of indirect paths. However, the size of the indirect effects was changed after adding the moderator. Specifically, the size of the indirect effects reduced, which confirms the existence of the moderated mediation model. These findings support the validity of the last hypothesis. However, the unfit indices were considered as problems with this model.

**Table 4:** Evaluation of the moderated mediation model.

Mediator Path	Indirect Path	Size of Effect (β)		Observed Natural	
		Before	After	Before	After
STE → RPBE → MPS	STE → RPBE	0.541	0.476	Significant Partial Mediation	Significant Partial Mediation
	RPBE → MPS	0.323	0.245		
SBT → RPBE → MPS	SBT → RPBE	-0.353	-0.431	Significant Partial Mediation	Significant Partial Mediation
	RPBE → MPS	0.323	0.245		

## 5 Discussion

**Fig. 8:**
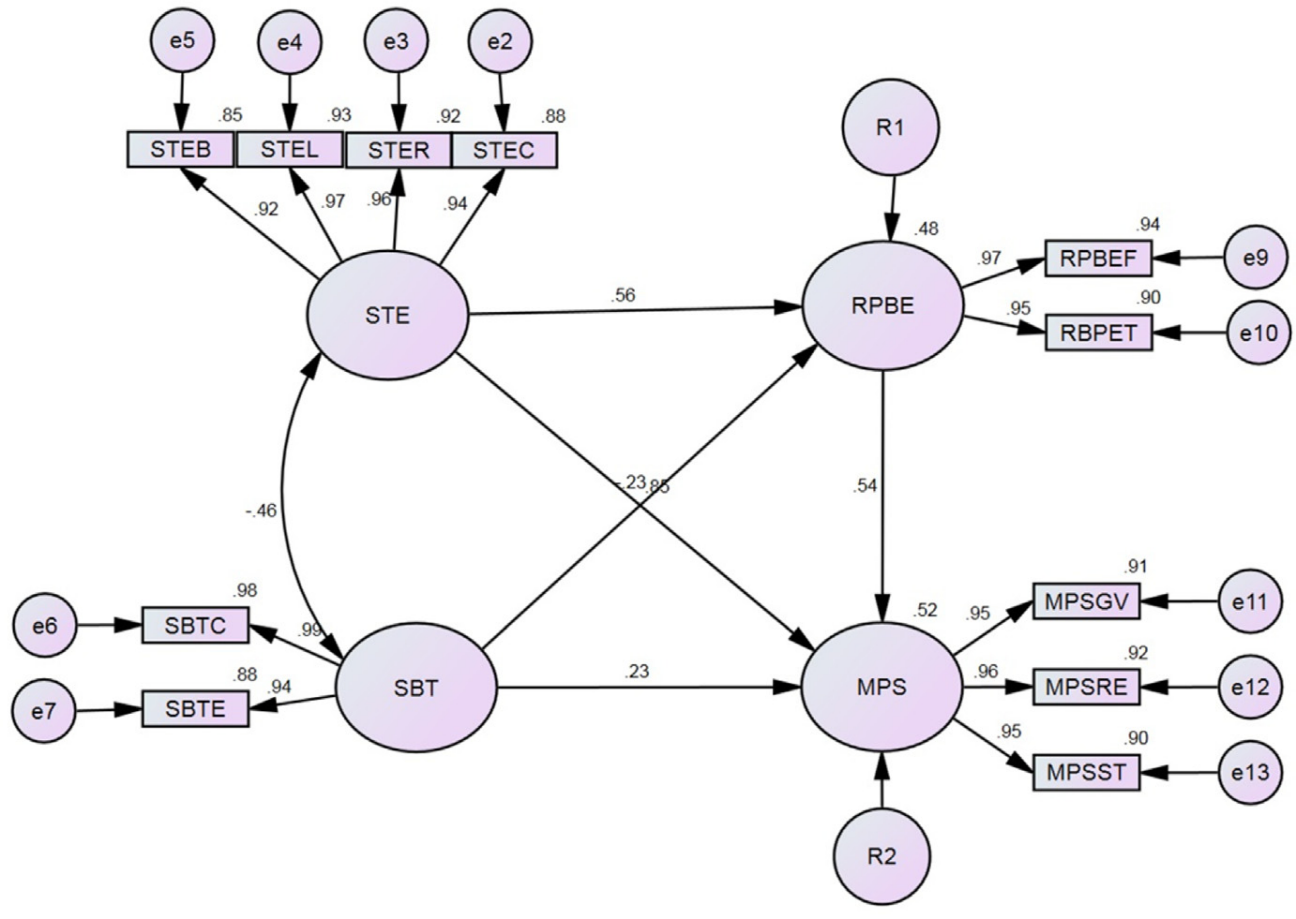
Graphical representation of the standardized estimates of the confirmed basic model.

The present paper successfully attributed about 50%–55% of the variance in the motivational problem of taking shortcuts was statistically and significantly explained by the combined effects of safety training effectiveness and stress-based tension when these factors are transmitted through routine perception-based errors. Fig. 8 is a graphic representation of the standardized estimates and proportion of variance for the confirmed basic structural model. Moreover, 48% of the variance in feeling and thinking about risk management was significantly explained by the same exogenous factors.

On the other hand, the same range of proportion of variance was observed in the alternative model when stress-based tension was moderated the causal path between safety training effectiveness and motivational problem of taking shortcuts. Unlike the first model, 73% of the variance in feeling and thinking about risk management was significantly explained in the alternative model. These findings were obtained when safety training effectiveness was influenced by the effects of a combination of different indicators such as coverage, reaction, learning, and behavior, which significantly explained 88%, 92%, 93%, and 83% of the variance, respectively, according to a cross-sectional study within three major cities in KSA.

Safety training improved 36.2% of safety perception (Pecquet, 2013) and 27.56% as reported by Von Thiele Schwarz et al. (2016). In the present paper, it could improve about 48%–73% when the work stressors are also under control. About 31.5% of the safety perceptions were explained up to 94% by feeling-based risks and 90% by thinking-based risks. The behavior explained 32% of safety perceptions as reported by Pecquet (2013), while it significantly explained 47.7% in the present study. The learning from safety training explained 22% of safety perception as highlighted by Bergheim et al. (2015), while 52.1% of the same was explained in the present study. However, the highlighted four dimensions of safety training have been statistically proved as vital issues in safety training effectiveness, which was not statistically clarified in previous studies.

Risk communication and physical work environment are main issues that contribute to work if they are based on rational logic. The effect of risk communication on safety perception explained 44.3% of the variance as reported by Hobbs and Williamson (2003), and the same significantly and negatively explained 22.54% of the variance in the routine perception-based error as presented in this paper. This indicates the current training programs in the selected construction sites were not covered the risk perception toward the risks associated with shortcuts. Ghahramani and Khalkhali (2015), highlighted 22.12% of the variance in safety perception was statistically explained by a combination of safety communication and supportive environment. In the present study, the same criterion explained 72.3% of the variance in the routine perception-based error. This confirms the effect of current work environment in the selected sites on perceiving the management risks.

However, the goodness fit indices in the alternative model were not supported by the present cross-sectional study in the scope of the selected environment and large sample size is required to confirm the model fit. Nevertheless, six independent indicators were identified in the theorized basic model, namely, coverage (skill, knowledge, and attitude), reaction (degree of interest or favorably to learn), learn (degree of acquiring appropriate skills, knowledge, and attitudes), behavior (degree of applying trained subjects), communication (degree of involvement and consumption of communication contents), and physical work environment (quality of the work environment, including hazards/controls). The first four indicators have been used to infer about the exogenous factor of safety training effectiveness. The remaining three indicators have been used to deduce about stress-based tension, another exogenous factor. Those two factors independently act and conversely affect the motivational problem of taking shortcuts (saving time, reducing effort, and gaining value) and they are partially transmitted through routine perception-based errors (feeling-based risks and thinking-based risks).

## 6 Conclusions

45%–55% of the total effects on risks associated with taking shortcuts were explained in the present study. Effectiveness of safety training and stress-based tension significantly explained a proportion of the variance when these exogenous factors were significantly and partially transmitted through routine perception-based errors. The significantly moderated mediation did not sustain the overall fit of the theorized alternative model. This can be considered as a recommended area for future work.

## Declarations

### Author contribution statement

Adel M. Al-Shayea, Mohamed Z. Ramadan, Khalid H. Al-Yami: Conceived and designed the experiments; Performed the experiments; Analyzed and interpreted the data; Contributed reagents, materials, analysis tools or data; Wrote the paper.

### Funding statement

This research did not receive any specific grant from funding agencies in the public, commercial, or not-for-profit sectors.

### Competing interest statement

The authors declare no conflict of interest.

### Additional information

No additional information is available for this paper.

## Appendix A Supplementary data

Supplemental file Supplemental file

Questionnaire Questionnaire
