# Supplementary material for: Structural model of factors contributing to the motivational problem of taking shortcuts at construction workplaces in the Kingdom of Saudi Arabia
Source: Heliyon. 2019 Feb 15;5(2):e01220. doi: 10.1016/j.heliyon.2019.e01220 (PMC6378347; doi:10.1016/j.heliyon.2019.e01220)
Supplement: Questionnaire [file mmc2.docx]

**Appendix: Final Design of Mailed Questionnaire**

Dear participant

Kindly, I am looking for your highly collaboration to accept my request, in answering the enclosed questionnaire. I am appreciating your time and effort for participating and answering this survey. This survey is part of a PhD study, conducted for measuring the effects of most possible factors associated with most occurrence type of unsafe acts (shortcuts) and the forefront type of construction accidents known as falling from height when the safe work-instruction is in place. It is a matter of less than 30 minutes from your-side to add a valuable information in research field. The scope of this questionnaire is to find the effects of different indicators to the motivational problems to use shortcuts (saving time, reducing effort or gaining value).

For your assurance all the data that will be collected through this survey are completely confidential. In addition, there are no recognizable risks in your answer to the questionnaire, as this study aims to highlight the highest effects to motivational problems to use shortcuts. I am hopeful that the results will contribute to clarify the perspective of improving the level of understanding the human needs leading to better safeties in construction sites.

Your participation in the questionnaire is voluntary and you may discontinue at any time.

Thank you for your time, interest, support and commitment to contribute to this research. Please, if you have any questions, contact me through my personal number: 00966503887101 (using WhatsApp) or through email (kaha-yami@al-babtain.com.sa).

Sincerely,

KHALID HASAN ALI AL-YAMI

King Saud University; Riyadh

Industrial Engineering College

Course: Doctor of Philosophy (PhD) in Human Factors and Safety

| I am appreciating your participation to answer 64 items in this questionnaire. I am posting my thanks in advance for your response to this survey. | | | | | | | | | | |  |  |  |
| --- | --- | --- | --- | --- | --- | --- | --- | --- | --- | --- | --- | --- | --- |
|  | | | | | | | | | | |  |  |  |
| Survey begins (your current achievement is 0% and you are able to reach 100%) | | | | | | | | | | |  |  |  |
| 1. Safety Training Effectiveness (inferred by five indicators as follows) | | | | | | | | | | |  |  |  |
|  | 1.1 **Frequency** (number of safety training and repeating the same program in single year) | | | | | | | | | | | | |
|  | | | |  | | | | | Number of safety training received in single year. | |  |  |  |
|  | | | |  | | | | | Number of other safety awareness programs given in single year | |  |  |  |
|  | | | |  | | | | | Number of same training program repeated in same year. | |  |  |  |
|  | | | |  | | | | | Number of same safety awareness programs repeated in same year. | |  |  |  |
|  | - 1. **Coverage** of require knowledge, skill and attitude in the giving safety program   (Just tick one answer from each question that is really explained your situation) | | | | | | | | | | | |  |
|  | 1.2.1 **The given safety training covers knowledge about risks of using shortcuts.** | | | | | | | | | | | |  |
|  |  | | | | |  | | | Strongly disagree | | | |  |
|  |  | | | | |  | | | Disagree | | | |  |
|  |  | | | | |  | | | Neutral | | | |  |
|  |  | | | | |  | | | Agree | | | |  |
|  |  | | | | |  | | | Strongly agree | | | |  |
| 1.2.2 **The given safety training covers skills to deal with risk when I expose to it.** | | | | | | | | | | |  |  |  |
|  | | | |  | | | | | Strongly disagree | |  |  |  |
|  | | | |  | | | | | Disagree | |  |  |  |
|  | | | |  | | | | | Neutral | |  |  |  |
|  | | | |  | | | | | Agree | |  |  |  |
|  | | | |  | | | | | Strongly agree | |  |  |  |
| Survey begins (your current achievement is 9% and you are able to reach 100%) | | | | | | | | | | |  |  |  |
| 1.2.3 **The given safety training provides clear information about chain of events related to risks of using shortcuts.** | | | | | | | | | | |  |  |  |
|  | | | |  | | | | | Strongly disagree | |  |  |  |
|  | | | |  | | | | | Disagree | |  |  |  |
|  | | | |  | | | | | Neutral | |  |  |  |
|  | | | |  | | | | | Agree | |  |  |  |
|  | | | |  | | | | | Strongly agree | |  |  |  |
|  | | | | | | | | | | |  |  |  |
| 1.2.4 **The given safety training explains management feelings and thoughts to human needs at workplace.** | | | | | | | | | | |  |  |  |
|  | | | |  | | | | | Strongly disagree | |  |  |  |
|  | | | |  | | | | | Disagree | |  |  |  |
|  | | | |  | | | | | Neutral | |  |  |  |
|  | | | |  | | | | | Agree | |  |  |  |
|  | | | |  | | | | | Strongly agree | |  |  |  |
|  |  | | | | | | | | | | | |  |
| 1.2.5 **Roles and responsibilities related to safe work practices and reporting near-miss are demonstrated through the safety training.** | | | | | | | | | | |  |  |  |
|  | | | |  | | | | | Strongly disagree | |  |  |  |
|  | | | |  | | | | | Disagree | |  |  |  |
|  | | | |  | | | | | Neutral | |  |  |  |
|  | | | |  | | | | | Agree | |  |  |  |
|  | | | |  | | | | | Strongly agree | |  |  |  |
|  | | | |  | | | | |  | |  |  |  |
|  | Survey continues (your current achievement is 14% and you are able to reach 100%) | | | | | | | | | | | |  |
|  | - 1. Your **reaction** to the given safety training   (Just tick one answer from each question that is really explained your situation) | | | | | | | | | | | |  |
|  | 1.3.1 **The given safety training is favored because it has explained the reality of my work-lives and all available risks in my workplace.** | | | | | | | | | | | |  |
|  |  | | | | |  | | | Strongly disagree | | | |  |
|  |  | | | | |  | | | Disagree | | | |  |
|  |  | | | | |  | | | Neutral | | | |  |
|  |  | | | | |  | | | Agree | | | |  |
|  |  | | | | |  | | | Strongly agree | | | |  |
| 1.3.2 **I and other trainees have satisfied from the given safety training that adds value on minimizing accidents in our workplace.** | | | | | | | | | | |  |  |  |
|  | | | |  | | | | | Strongly disagree | |  |  |  |
|  | | | |  | | | | | Disagree | |  |  |  |
|  | | | |  | | | | | Neutral | |  |  |  |
|  | | | |  | | | | | Agree | |  |  |  |
|  | | | |  | | | | | Strongly agree | |  |  |  |
| 1.3.3 **I and my colleagues like to attend every safety training to understand our feeling about risks of using shortcuts.** | | | | | | | | | | |  |  |  |
|  | | | |  | | | | | Strongly disagree | |  |  |  |
|  | | | |  | | | | | Disagree | |  |  |  |
|  | | | |  | | | | | Neutral | |  |  |  |
|  | | | |  | | | | | Agree | |  |  |  |
|  | | | |  | | | | | Strongly agree | |  |  |  |
|  | | | |  | | | | |  | |  |  |  |
| Survey continues (your current achievement is 19% and you are able to reach 100%) | | | | | | | | | | |  |  |  |
| 1.3.4 **The given safety training contains interesting subjects to accommodate between my work and life.** | | | | | | | | | | |  |  |  |
|  | | | |  | | | | | Strongly disagree | |  |  |  |
|  | | | |  | | | | | Disagree | |  |  |  |
|  | | | |  | | | | | Neutral | |  |  |  |
|  | | | |  | | | | | Agree | |  |  |  |
|  | | | |  | | | | | Strongly agree | |  |  |  |
| 1.3.5 **If I asked to evaluate the given safety training to me, the overall evaluation is always SMILED FACE** ☺ | | | | | | | | | | |  |  |  |
|  | | | |  | | | | | Strongly disagree | |  |  |  |
|  | | | |  | | | | | Disagree | |  |  |  |
|  | | | |  | | | | | Neutral | |  |  |  |
|  | | | |  | | | | | Agree | |  |  |  |
|  | | | |  | | | | | Strongly agree | |  |  |  |
|  |  |  |  |  |  |  |  |  |  |  |  |  |  |
|  |  |  |  |  |  |  |  |  |  |  |  |  |  |
| Survey continues (your current achievement is 22% and you are able to reach 100%) | | | | | | | | | | |  |  |  |
|  | - 1. **Leaning** from the given safety training   (Just tick one answer from each question that is really explained your situation) | | | | | | | | | | | |  |
|  | 1.4.1 **I gain valuable knowledge and skills from the given safety training to deal positively when I observe unsafe work behaviour.** | | | | | | | | | | | |  |
|  |  | | | | |  | | | Strongly disagree | | | |  |
|  |  | | | | |  | | | Disagree | | | |  |
|  |  | | | | |  | | | Neutral | | | |  |
|  |  | | | | |  | | | Agree | | | |  |
|  |  | | | | |  | | | Strongly agree | | | |  |
| 1.4.2 **Sometime, I have negative attitude because of work circumstances but I learn from safety training the best way to mitigate that attitude positively.** | | | | | | | | | | |  |  |  |
|  | | | |  | | | | | Strongly disagree | |  |  |  |
|  | | | |  | | | | | Disagree | |  |  |  |
|  | | | |  | | | | | Neutral | |  |  |  |
|  | | | |  | | | | | Agree | |  |  |  |
|  | | | |  | | | | | Strongly agree | |  |  |  |
| 1.4.3 **After safety training, I have learned how to make work-life balancing.** | | | | | | | | | | |  |  |  |
|  | | | |  | | | | | Strongly disagree | |  |  |  |
|  | | | |  | | | | | Disagree | |  |  |  |
|  | | | |  | | | | | Neutral | |  |  |  |
|  | | | |  | | | | | Agree | |  |  |  |
|  | | | |  | | | | | Strongly agree | |  |  |  |
|  | | | | | | | | | | |  |  |  |
| Survey continues (your current achievement is 27 % and you are able to reach 100%) | | | | | | | | | | |  |  |  |
| 1.4.4 **The given safety training shows us how to report cases of unsafe work behaviour and effectively response to receive report of similar cases.** | | | | | | | | | | |  |  |  |
|  | | | |  | | | | | Strongly disagree | |  |  |  |
|  | | | |  | | | | | Disagree | |  |  |  |
|  | | | |  | | | | | Neutral | |  |  |  |
|  | | | |  | | | | | Agree | |  |  |  |
|  | | | |  | | | | | Strongly agree | |  |  |  |
| 1.4.5 **Safety training does not help much to learn about risk management.** | | | | | | | | | | |  |  |  |
|  | | | |  | | | | | Strongly disagree | |  |  |  |
|  | | | |  | | | | | Disagree | |  |  |  |
|  | | | |  | | | | | Neutral | |  |  |  |
|  | | | |  | | | | | Agree | |  |  |  |
|  | | | |  | | | | | Strongly agree | |  |  |  |
|  | - 1. **Behaviour** from the given safety training as it is useful to do what you have learned in your work (Just tick one answer from each question that is really explained your situation) | | | | | | | | | | | |  |
|  | 1.5.1 **I and my colleagues believe that the safety training is essential for continual improvement to sustain safe work practices.** | | | | | | | | | | | |  |
|  |  | | | | |  | | | Strongly disagree | | | |  |
|  |  | | | | |  | | | Disagree | | | |  |
|  |  | | | | |  | | | Neutral | | | |  |
|  |  | | | | |  | | | Agree | | | |  |
|  |  | | | | |  | | | Strongly agree | | | |  |
|  | | | | | | | | | | |  |  |  |
| Survey continues (your current achievement is 31% and you are able to reach 100%) | | | | | | | | | | |  |  |  |
| 1.5.2 **My knowledge and skills have been improved after safety training.** | | | | | | | | | | |  |  |  |
|  | | | |  | | | | | Strongly disagree | |  |  |  |
|  | | | |  | | | | | Disagree | |  |  |  |
|  | | | |  | | | | | Neutral | |  |  |  |
|  | | | |  | | | | | Agree | |  |  |  |
|  | | | |  | | | | | Strongly agree | |  |  |  |
|  | | | | | | | | | | |  |  |  |
| 1.5.3 **I and my work-team have believed that the positive work behaviour can be achieved through the safety training.** | | | | | | | | | | |  |  |  |
|  | | | |  | | | | | Strongly disagree | |  |  |  |
|  | | | |  | | | | | Disagree | |  |  |  |
|  | | | |  | | | | | Neutral | |  |  |  |
|  | | | |  | | | | | Agree | |  |  |  |
|  | | | |  | | | | | Strongly agree | |  |  |  |
|  | | | | | | | | | | |  |  |  |
| 1.5.4 **Safety is always in the first priority in my work and it becomes my habit after attending several safety training.** | | | | | | | | | | |  |  |  |
|  | | | |  | | | | | Strongly disagree | |  |  |  |
|  | | | |  | | | | | Disagree | |  |  |  |
|  | | | |  | | | | | Neutral | |  |  |  |
|  | | | |  | | | | | Agree | |  |  |  |
|  | | | |  | | | | | Strongly agree | |  |  |  |
|  | | | | | | | | | | |  |  |  |
| Survey continues (your current achievement is 36% and you are able to reach 100%) | | | | | | | | | | |  |  |  |
| 1.5.5 **In my workplace, I can report any unsafe work practice to my supervisor.** | | | | | | | | | | |  |  |  |
|  | | | |  | | | | | Strongly disagree | |  |  |  |
|  | | | |  | | | | | Disagree | |  |  |  |
|  | | | |  | | | | | Neutral | |  |  |  |
|  | | | |  | | | | | Agree | |  |  |  |
|  | | | |  | | | | | Strongly agree | |  |  |  |
|  |  | | | | | | | | | | | |  |
|  | Comment about safety training effectiveness (*optional*): ……………..……………………. | | | | | | | | | | | |  |
|  | ……………………………………………………………………….…………………….. | | | | | | | | | | | |  |
|  | ……………………………………………………………………….…………………….. | | | | | | | | | | | |  |
|  | ……………………………………………………………………….…………………….. | | | | | | | | | | | |  |
|  | ……………………………………………………………………….…………………….. | | | | | | | | | | | |  |
|  | ……………………………………………………………………….…………………….. | | | | | | | | | | | |  |
|  | ……………………………………………………………………….…………………….. | | | | | | | | | | | |  |
|  | ……………………………………………………………………….…………………….. | | | | | | | | | | | |  |
|  | ……………………………………………………………………….…………………….. | | | | | | | | | | | |  |
|  | ……………………………………………………………………….…………………….. | | | | | | | | | | | |  |
|  | ……………………………………………………………………….…………………….. | | | | | | | | | | | |  |
|  | ……………………………………………………………………….…………………….. | | | | | | | | | | | |  |
|  | ……………………………………………………………………….…………………….. | | | | | | | | | | | |  |
| Survey continues (your current achievement is 38% and you are able to reach 100%) | | | | | | | | | | | | |  |
| 1. Stress-based Tension (inferred by three indicators as follows) | | | | | | | | | | |  |  |  |
|  | 2.1 **Communication** (from workers to leaders and vice versa)  (Just tick one answer from each question that is really explained your situation) | | | | | | | | | | | |  |
|  | 2.1.1 **In my workplace, I have never been selected as an active member in communication contents and my opinions about safety was not considered.** | | | | | | | | | | | |  |
|  |  | | | | |  | | | Strongly disagree | | | |  |
|  |  | | | | |  | | | Disagree | | | |  |
|  |  | | | | |  | | | Neutral | | | |  |
|  |  | | | | |  | | | Agree | | | |  |
|  |  | | | | |  | | | Strongly agree | | | |  |
| 2.1.2 **The leadership does not pay-attention to my needs and not given the valuable feedback.** | | | | | | | | | | |  |  |  |
|  | | | |  | | | | | Strongly disagree | |  |  |  |
|  | | | |  | | | | | Disagree | |  |  |  |
|  | | | |  | | | | | Neutral | |  |  |  |
|  | | | |  | | | | | Agree | |  |  |  |
|  | | | |  | | | | | Strongly agree | |  |  |  |
| 2.1.3 **The safe work-instruction violation is a result of ignoring the highlighted unsafe work behaviour.** | | | | | | | | | | | | |  |
|  | | | | |  | | | | | Strongly disagree | | |  |
|  | | | | |  | | | | | Disagree | | |  |
|  | | | | |  | | | | | Neutral | | |  |
|  | | | | |  | | | | | Agree | | |  |
|  | | | | |  | | | | | Strongly agree | | |  |
|  | | | | | | | | | | | | |  |
| Survey continues (your current achievement is 42% and you are able to reach 100%) | | | | | | | | | | | | |  |
| 2.1.4 **No well communication to control employees or getting solution to those employees.** | | | | | | | | | | | | |  |
|  | | | | |  | | | | | Strongly disagree | | |  |
|  | | | | |  | | | | | Disagree | | |  |
|  | | | | |  | | | | | Neutral | | |  |
|  | | | | |  | | | | | Agree | | |  |
|  | | | | |  | | | | | Strongly agree | | |  |
| 2.1.5 **Present communication leads to undesirable work relationship, which in turn affects the safety performance.** | | | | | | | | | | | | |  |
|  | | | | |  | | | | | Strongly disagree | | |  |
|  | | | | |  | | | | | Disagree | | |  |
|  | | | | |  | | | | | Neutral | | |  |
|  | | | | |  | | | | | Agree | | |  |
|  | | | | |  | | | | | Strongly agree | | |  |
|  | 2.2 **Physical Work Environment** (risk exposure in your workplace)  (Just tick one answer from each question that is really explained your situation) | | | | | | | | | | | |  |
|  | 2.2.1 **The frequent risk exposure is a result of accepting unsafe work behaviour in my workplace.** | | | | | | | | | | | |  |
|  |  | | | | |  | | | Strongly disagree | | | |  |
|  |  | | | | |  | | | Disagree | | | |  |
|  |  | | | | |  | | | Neutral | | | |  |
|  |  | | | | |  | | | Agree | | | |  |
|  |  | | | | |  | | | Strongly agree | | | |  |
|  | | | | | | | | | | | | |  |
| Survey continues (your current achievement is 47% and you are able to reach 100%) | | | | | | | | | | | | |  |
| 2.2.2 **The surrounding work environment does not improve and it increases our tension.** | | | | | | | | | | | | |  |
|  | |  | | | | | Strongly disagree | | | | |  |  |
|  | |  | | | | | Disagree | | | | |  |  |
|  | |  | | | | | Neutral | | | | |  |  |
|  | |  | | | | | Agree | | | | |  |  |
|  | |  | | | | | Strongly agree | | | | |  |  |
|  | | | | | | | | | | | | |  |
| 2.2.3 **Working under tension is a result of safety violation in my workplace.** | | | | | | | | | | | | |  |
|  | | |  | | | | | Strongly disagree | | | | |  |
|  | | |  | | | | | Disagree | | | | |  |
|  | | |  | | | | | Neutral | | | | |  |
|  | | |  | | | | | Agree | | | | |  |
|  | | |  | | | | | Strongly agree | | | | |  |
| 2.2.4 **Site leadership does not control sources of tension to enhance the safe work environment in my workplace.** | | | | | | | | | | | | |  |
|  | | |  | | | | | Strongly disagree | | | | |  |
|  | | |  | | | | | Disagree | | | | |  |
|  | | |  | | | | | Neutral | | | | |  |
|  | | |  | | | | | Agree | | | | |  |
|  | | |  | | | | | Strongly agree | | | | |  |
|  | | | | | | | | | | | | |  |
| Survey continues (your current achievement is 52% and you are able to reach 100%) | | | | | | | | | | | | |  |
| 2.2.5 **The present environmental condition does not promote for safe work practices.** | | | | | | | | | | | | |  |
|  | | |  | | | | | Strongly disagree | | | | |  |
|  | | |  | | | | | Disagree | | | | |  |
|  | | |  | | | | | Neutral | | | | |  |
|  | | |  | | | | | Agree | | | | |  |
|  | | |  | | | | | Strongly agree | | | | |  |
|  | 2.3 **Margin of Personal Life-time** (the available time and time for your needs)  (Just tick one answer from each question that is really explained your situation) | | | | | | | | | | | |  |
|  | 2.3.1 **The available time does not help to make balancing between the required works and my personal issues.** | | | | | | | | | | | |  |
|  |  | | | | |  | | | Strongly disagree | | | |  |
|  |  | | | | |  | | | Disagree | | | |  |
|  |  | | | | |  | | | Neutral | | | |  |
|  |  | | | | |  | | | Agree | | | |  |
|  |  | | | | |  | | | Strongly agree | | | |  |
|  | 2.3.2 **I like to reduce the working time to have time for achieving my personal issues.** | | | | | | | | | | | |  |
|  |  | | | | |  | | | Strongly disagree | | | |  |
|  |  | | | | |  | | | Disagree | | | |  |
|  |  | | | | |  | | | Neutral | | | |  |
|  |  | | | | |  | | | Agree | | | |  |
|  |  | | | | |  | | | Strongly agree | | | |  |

| Survey continues (your current achievement is 56% and you are able to reach 100%) | | | | | | | | | | |  |  |  |
| --- | --- | --- | --- | --- | --- | --- | --- | --- | --- | --- | --- | --- | --- |
| 2.3.3 **My tension always increases because I cannot reach my personal issues.** | | | | | | | | | | | | |  |
|  | | |  | | | Strongly disagree | | | | | | |  |
|  | | |  | | | Disagree | | | | | | |  |
|  | | |  | | | Neutral | | | | | | |  |
|  | | |  | | | Agree | | | | | | |  |
|  | | |  | | | Strongly agree | | | | | | |  |
| 2.3.4 **My personal issues become routine activities that always need time.** | | | | | | | | | | | | |  |
|  | | |  | | | Strongly disagree | | | | | | |  |
|  | | |  | | | Disagree | | | | | | |  |
|  | | |  | | | Neutral | | | | | | |  |
|  | | |  | | | Agree | | | | | | |  |
|  | | |  | | | Strongly agree | | | | | | |  |
| 2.3.5 **My works are well scheduled and I have more flexible working time.** | | | | | | | | | | | | |  |
|  | | |  | | | Strongly disagree | | | | | | |  |
|  | | |  | | | Disagree | | | | | | |  |
|  | | |  | | | Neutral | | | | | | |  |
|  | | |  | | | Agree | | | | | | |  |
|  | | |  | | | Strongly agree | | | | | | |  |
|  | | | | | | | | | | | | |  |
| Comment about tension-based stress (if any): …………………...……………………….......  …………………………………………………………………………………………………………………………………………………………………………………………………………………… | | | | | | | | | | | | |  |
|  | | | | | | | | | | |  |  |  |
| Survey continues (your current achievement is 61% and you are able to reach 100%) | | | | | | | | | | |  |  |  |
| 1. Routine Perception-based Error (inferred by two indicators as follows) | | | | | | | | | | |  |  |  |
|  | 3.1 **Feeling-based Risk** (your consideration when you expose to risk)  (Just tick one answer from each question that is really explained your situation) | | | | | | | | | | | |  |
|  | 3.1.1 **I feel safety training refreshment will support trainees to retrieve the gained knowledge for better understanding.** | | | | | | | | | | | |  |
|  |  |  | | | | | Strongly disagree | | |  |  |  |  |
|  |  |  | | | | | Disagree | | |  |  |  |  |
|  |  |  | | | | | Neutral | | |  |  |  |  |
|  |  |  | | | | | Agree | | |  |  |  |  |
|  |  |  | | | | | Strongly agree | | |  |  |  |  |
|  | 3.1.2 **I feel worker engagement in determination of safety training subjects will help to safety compliance.** | | | | | | | | | | | |  |
|  |  | | |  | | | | Strongly disagree | | | | |  |
|  |  | | |  | | | | Disagree | | | | |  |
|  |  | | |  | | | | Neutral | | | | |  |
|  |  | | |  | | | | Agree | | | | |  |
|  |  | | |  | | | | Strongly agree | | | | |  |
|  | 3.1.3 **I feel that well communication increases our understanding to the requirements and in turn contributes to good achievement.** | | | | | | | | | | | |  |
|  |  | | |  | | | | Strongly disagree | | | | |  |
|  |  | | |  | | | | Disagree | | | | |  |
|  |  | | |  | | | | Neutral | | | | |  |
|  |  | | |  | | | | Agree | | | | |  |
|  |  | | |  | | | | Strongly agree | | | | |  |
|  |  | | |  | | | |  | | | | |  |
|  | Survey continues (your current achievement is 66% and you are able to reach 100%) | | | | | | | | | | | |  |
|  | 3.1.4 **I tolerate some unsafe work behaviour when I feel my workplace environment will not be well maintained.** | | | | | | | | | | | |  |
|  |  | | |  | | | | Strongly disagree | | | | |  |
|  |  | | |  | | | | Disagree | | | | |  |
|  |  | | |  | | | | Neutral | | | | |  |
|  |  | | |  | | | | Agree | | | | |  |
|  |  | | |  | | | | Strongly agree | | | | |  |
|  | 3.1.5 **I reasonably speed-up my work to finish early when I feel there is no enough time to serve my important life-issues.** | | | | | | | | | | | |  |
|  |  | | |  | | | | Strongly disagree | | | | |  |
|  |  | | |  | | | | Disagree | | | | |  |
|  |  | | |  | | | | Neutral | | | | |  |
|  |  | | |  | | | | Agree | | | | |  |
|  |  | | |  | | | | Strongly agree | | | | |  |
|  | 3.2 **Thinking-based Risk** (logic way to decide when you expose to risk)  (Just tick one answer from each question that is really explained your situation) | | | | | | | | | | | |  |
|  | 3.2.1 **I think safety training without refreshment to the same training will lead to loss the gained knowledge for making safe work behaviour.** | | | | | | | | | | | |  |
|  |  | | |  | | | | Strongly disagree | | | | |  |
|  |  | | |  | | | | Disagree | | | | |  |
|  |  | | |  | | | | Neutral | | | | |  |
|  |  | | |  | | | | Agree | | | | |  |
|  |  | | |  | | | | Strongly agree | | | | |  |
|  |  | | |  | | | |  | | | | |  |
|  | Survey continues (your current achievement is 70% and you are able to reach 100%) | | | | | | | | | | | |  |
| 3.2.2 **I can work in safe manner if site leadership will listen to my complaint about the source of tension in my workplace and promoting solution to me.** | | | | | | | | | | | | |  |
|  | | |  | | | Strongly disagree | | | | | | |  |
|  | | |  | | | Disagree | | | | | | |  |
|  | | |  | | | Neutral | | | | | | |  |
|  | | |  | | | Agree | | | | | | |  |
|  | | |  | | | Strongly agree | | | | | | |  |
|  | 3.2.3 **I think if my workplace is maintained good work environment, it will help workers to see and report risks.** | | | | | | | | | | | |  |
|  |  | | |  | | | | Strongly disagree | | | | |  |
|  |  | | |  | | | | Disagree | | | | |  |
|  |  | | |  | | | | Neutral | | | | |  |
|  |  | | |  | | | | Agree | | | | |  |
|  |  | | |  | | | | Strongly agree | | | | |  |
|  |  | | | | | | | | | | | | |
|  | 3.2.4 **I think the only way to reach safety compliance is to maintain effective communication among employees.** | | | | | | | | | | | |  |
|  |  | | |  | | | | Strongly disagree | | | | |  |
|  |  | | |  | | | | Disagree | | | | |  |
|  |  | | |  | | | | Neutral | | | | |  |
|  |  | | |  | | | | Agree | | | | |  |
|  |  | | |  | | | | Strongly agree | | | | |  |
|  |  | | |  | | | |  | | | | |  |
|  | Survey continues (your current achievement is 75% and you are able to reach 100%) | | | | | | | | | | | | |
|  | 3.2.5 **I think given time for doing my personal issues will increase my work commitment and reduce violation.** | | | | | | | | | | | | |
|  |  | | | |  | | | | Strongly disagree | | | | |
|  |  | | | |  | | | | Disagree | | | | |
|  |  | | | |  | | | | Neutral | | | | |
|  |  | | | |  | | | | Agree | | | | |
|  |  | | | |  | | | | Strongly agree | | | | |
|  | | | | | | | | | | | | |  |
| Comment about routine perception-based error (if any): ……………….……………….......  …………………………………………………………………………………………………………………………….……………………………………………………………………………………………………………………………………………………………………………………………… | | | | | | | | | | | | |  |
| 1. Motivational Problems to Shortcuts | | | | | | | | | | | |  |  |
|  | 4.1 **Saving Time** (doing the work quickly without following instruction to save time for the following situations)  (Just tick one answer from each question that is really explained your situation) | | | | | | | | | | | | |
|  | 4.1.1 **I am working full time and breaking-time is only allowed free time to serve my needs.** | | | | | | | | | | | | |
|  |  | | | |  | | | | Strongly disagree | | | | |
|  |  | | | |  | | | | Disagree | | | | |
|  |  | | | |  | | | | Neutral | | | | |
|  |  | | | |  | | | | Agree | | | | |
|  |  | | | |  | | | | Strongly agree | | | | |
|  |  | | | | | | | | | | | | |
|  | Survey continues (your current achievement is 78% and you are able to reach 100%) | | | | | | | | | | | | |
|  | 4.1.2 **There is no chance in my workplace to get permission for doing some personal issues out of my break-time.** | | | | | | | | | | | | |
|  |  | | | |  | | | | Strongly disagree | | | | |
|  |  | | | |  | | | | Disagree | | | | |
|  |  | | | |  | | | | Neutral | | | | |
|  |  | | | |  | | | | Agree | | | | |
|  |  | | | |  | | | | Strongly agree | | | | |
|  |  | | | | | | | | | | | | |
|  | 4.1.3 **I have to achieve my work fast to save time for doing my personal issues.** | | | | | | | | | | | | |
|  |  | | | |  | | | | Strongly disagree | | | | |
|  |  | | | |  | | | | Disagree | | | | |
|  |  | | | |  | | | | Neutral | | | | |
|  |  | | | |  | | | | Agree | | | | |
|  |  | | | |  | | | | Strongly agree | | | | |
|  |  | | | | | | | | | | | | |
|  | 4.1.4 **I like to follow safety issues but there is no sufficient time to do my work with full safety requirements.** | | | | | | | | | | | | |
|  |  | | | |  | | | | Strongly disagree | | | | |
|  |  | | | |  | | | | Disagree | | | | |
|  |  | | | |  | | | | Neutral | | | | |
|  |  | | | |  | | | | Agree | | | | |
|  |  | | | |  | | | | Strongly agree | | | | |
|  | Survey continues (your current achievement is 83% and you are able to reach 100%) | | | | | | | | | | | | |
|  | 4.1.5 **Short time will increase my tension if I have urgent personal issue.** | | | | | | | | | | | | |
|  |  | | | |  | | | | Strongly disagree | | | | |
|  |  | | | |  | | | | Disagree | | | | |
|  |  | | | |  | | | | Neutral | | | | |
|  |  | | | |  | | | | Agree | | | | |
|  |  | | | |  | | | | Strongly agree | | | | |

|  | 4.2 **Reducing Effort** (doing the work quickly without following instruction when I have one of the following situations)  (Just tick one answer from each question that is really explained your situation) | | | | | | | |
| --- | --- | --- | --- | --- | --- | --- | --- | --- |
|  | 4.2.1 **I am always facing either sweaty or breathing heavily when I work up to the official break-time (tighten work schedule).** | | | | | | | |
|  |  | |  | | Strongly disagree | | | |
|  |  | |  | | Disagree | | | |
|  |  | |  | | Neutral | | | |
|  |  | |  | | Agree | | | |
|  |  | |  | | Strongly agree | | | |
|  |  | | | | | | | |
|  | 4.2.2 **The present work condition in my workplace is hurting and physically discomfort.** | | | | | | | |
|  |  | |  | | Strongly disagree | | | |
|  |  | |  | | Disagree | | | |
|  |  | |  | | Neutral | | | |
|  |  | |  | | Agree | | | |
|  |  | |  | | Strongly agree | | | |
|  |  | |  | |  | | | |
|  | Survey continues (your current achievement is 88% and you are able to reach 100%) | | | | | | | |
|  | 4.2.3 **I never become sweaty in my daily work and there is no need to have frequent break-times.** | | | | | | | |
|  |  | |  | | Strongly disagree | | | |
|  |  | |  | | Disagree | | | |
|  |  | |  | | Neutral | | | |
|  |  | |  | | Agree | | | |
|  |  | |  | | Strongly agree | | | |
|  | 4.2.4 **The available tool and equipment require more effort whereas the work can be manually achieved with less effort.** | | | | | | | |
|  |  | |  | | Strongly disagree | | | |
|  |  | |  | | Disagree | | | |
|  |  | |  | | Neutral | | | |
|  |  | |  | | Agree | | | |
|  |  | |  | | Strongly agree | | | |
|  |  | | | | | | | |
|  | 4.2.5 **I skip the available risk’s control when I feel it is required more effort.** | | | | | | | |
|  |  | |  | | Strongly disagree | | | |
|  |  | |  | | Disagree | | | |
|  |  | |  | | Neutral | | | |
|  |  | |  | | Agree | | | |
|  |  | |  | | Strongly agree | | | |
|  |  | |  | |  | | | |
|  | Survey continues (your current achievement is 92% and you are able to reach 100%) | | | | | | | |
|  | 4.3 Gain Value (doing the work quickly without following instruction when the following situations occur)  (Just tick one answer from each question that is really explained your situation) | | | | | | | |
|  | 4.3.1 **I will do more than my capability when my boss is appreciating me in front of others.** | | | | | | | |
|  |  | |  | | Strongly disagree | | | |
|  |  | |  | | Disagree | | | |
|  |  | |  | | Neutral | | | |
|  |  | |  | | Agree | | | |
|  |  | |  | | Strongly agree | | | |
|  | 4.3.2 **Given priority to achieve management needs and reduce effort for doing anything out of management interest.** | | | | | | | |
|  |  | |  | | Strongly disagree | | | |
|  |  | |  | | Disagree | | | |
|  |  | |  | | Neutral | | | |
|  |  | |  | | Agree | | | |
|  |  | |  | | Strongly agree | | | |
|  |  | | | | | | | |
|  | 4.3.3 **I do not like the untrustworthy promises from my manager and that is leading to poor work environment.** | | | | | | | |
|  |  | |  | | Strongly disagree | | | |
|  |  | |  | | Disagree | | | |
|  |  | |  | | Neutral | | | |
|  |  | |  | | Agree | | | |
|  |  | |  | | Strongly agree | | | |
|  |  | |  | |  | | | |
|  | Survey continues (your current achievement is 97% and you are valuable gentleman) | | | | | | | |
|  | 4.3.4 **In my workplace, workforce has motivated safety violator.** | | | | | | | |
|  |  | |  | | Strongly disagree | | | |
|  |  | |  | | Disagree | | | |
|  |  | |  | | Neutral | | | |
|  |  | |  | | Agree | | | |
|  |  | |  | | Strongly agree | | | |
|  | 4.3.5 **Continuous verbal motivation is better than discrete incentives.** | | | | | | | |
|  |  | |  | | Strongly disagree | | | |
|  |  | |  | | Disagree | | | |
|  |  | |  | | Neutral | | | |
|  |  | |  | | Agree | | | |
|  |  | |  | | Strongly agree | | | |
| Comment about shortcuts (if any): …………………………………….…………………....  ………………………………………………………………….………………………………………………………………………………………………………………………………………………………………………………………………………………………………………………………………………………………………………………………………………………………………………………………………………………………………………………………………………………………………………………………………………………………………………………………… | | | | | | | |  |
|  | Survey continues (your current achievement is 100% and you are valuable gentleman) | | | | | | | |
|  | \|  \| \| \| \| \| \| \| \| \| \| \| \| --- \| --- \| --- \| --- \| --- \| --- \| --- \| --- \| --- \| --- \| --- \| \| I am appreciating your answering 64 items in this questionnaire. I am posting my thanks again for your response to this survey. Please, provide us the following information: \| \| \| \| \| \| \| \| \| \| \| \|  \| \| \| \| \| \| \| \| \| \| \| \| Initial information about valuable participant (Just tick one box) \| \| \| \| \| \| \| \| \| \| \| \|  \| 1. **Type of current work field:** \| \| \| \| \| \| \| \| \| \| \| \|  \|  \|  \| Construction \| \|  \| Industrial \| \|  \| Other \| \| \| \| \|  \| Type of work (*optional*): …………………………………………………………. \| \| \| \| \| \| \| \| \| \| \| \|  \| 1. **Tick your location where you have been working:** \| \| \| \| \| \| \| \| \| \| \| \|  \|  \|  \| Riyadh \| \|  \| Dammam \| \| \| \| \| \| \| \|  \|  \|  \| Jeddah \| \|  \| Other city \| \| \| \| \| \| \| \|  \| 1. **Age:** \| \| \| \| \| \| \| \| \| \| \| \|  \|  \|  \| Below 30 years \| \| \|  \| From 30 to 60 years \| \| \| \| \| \| \|  \|  \|  \| Above 60 years \| \| \| \| \| \| \| \| \| \| \|  \| 1. **Qualification Level:** \| \| \| \| \| \| \| \| \| \| \| \|  \|  \|  \| No qualification \| \|  \| Less than Diploma \| \| \| \| \| \| \| \|  \|  \|  \| Diploma / Bachelor \| \|  \| Higher than Bachelor \| \| \| \| \| \| \| \|  \| 1. **Does your company certified in OHSAS 18001 or equivalent?** \| \| \| \| \| \| \| \| \| \| \| \|  \|  \|  \| Yes \| \|  \| No \| \| \| \| \| \| \| \|  \| 1. **Type of communication or receiving instruction at workplace:** \| \| \| \| \| \| \| \| \| \| \| \|  \|  \|  \| Verbal \| \|  \| Written \| \|  \| Both \| \| \| \| \|  \| 1. **How many years your experiences in the current work?** \| \| \| \| \| \| \| \| \| \| \| \|  \|  \|  \| Less than 1 year \|  \| \| Less than 5 years \| \| \| \|  \| \| \|  \|  \|  \| Less than 10 years \|  \| \| Above 10 years \| \| \| \|  \| \| \|  \| 1. **Your current designation:** \| \| \| \| \| \| \| \| \| \| \| \|  \|  \|  \| Manager / Leadership \| \|  \| Section Head / Site Engineer \| \| \| \| \| \| \| \|  \|  \|  \| Supervisor / Inspector \| \|  \| Worker / Technician \| \| \| \| \| \| \| \|  \| Other designation (*if any*): …………….………………………………………………. \| \| \| \| \| \| \| \| \| \| \| \|  \| 1. **Which one of following is your most interest:** (you can select both if you like all) \| \| \| \| \| \| \| \| \| \| \| \|  \|  \|  \| Political debate \| \|  \| Poetry \| \| \| \| \| \| \|  \|  \| 1. **Which one of following you like more:** (you can select both if you like all) \| \| \| \| \| \| --- \| --- \| --- \| --- \| --- \| --- \| \|  \|  \|  \| Math \|  \| Art \| | | | | | | | |
|  | 1. **Please, tick one of the following as your evaluation for the significant of the above question in your workplace.** | | | | | | | |
|  |  |  | | Not important | |  | Less important | |
|  |  |  | | Important | |  | Extremely important | |
|  | This an optional space to add any comment about the aforementioned survey: …………… | | | | | | | |
|  | ………………………………………………………………….………………………….. | | | | | | | |
|  | ………………………………………………………………….………………………….. | | | | | | | |
|  | ………………………………………………………………….………………………….. | | | | | | | |
|  | ………………………………………………………………….………………………….. | | | | | | | |
|  | 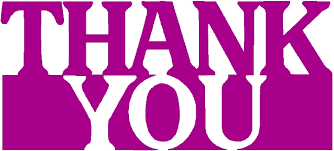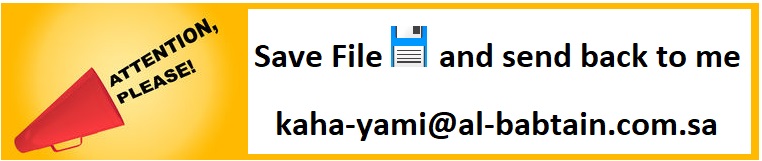 | | | | | | | |
